# Changes in BOLD variability are linked to the development of variable response inhibition

**DOI:** 10.1016/j.neuroimage.2020.117691

**Published:** 2021-03

**Authors:** Abigail Thompson, Margot A. Schel, Nikolaus Steinbeis

**Affiliations:** a Department of Clinical, Educational and Health Psychology, UCL, 26 Bedford Way, London WC1H 0AP, UK; b Institute of Education and Child Studies, Leiden University, 2333 AK, Leiden, the Netherlands

**Keywords:** Bold variability, SSRT, Response inhibition, Ex-Gaussian

## Abstract

Research on the development of response inhibition in humans has focused almost exclusively on average stopping performance. The development of intra-individual variability in stopping performance and its underlying neural circuitry has remained largely unstudied, even though understanding variability is of core importance for understanding development. In a total sample of 45 participants (19 children aged 10–12 years and 26 adults aged 18–26 years) of either sex we aimed to identify age-related changes in intra-individual response inhibition performance and its underlying brain signal variability. While there was no difference in average stopping performance between children and adults, stop signal latencies for the children were more variable. Further, brain signal variability during successful stopping was significantly higher in adults compared to children, especially in bilateral thalamus, but also across regions of the inhibition network. Finally, brain signal variability was significantly associated with stopping performance behavioral variability in adults. Together these results indicate that variability in stopping performance decreases, whereas neural variability in the inhibition network increases, from childhood to adulthood. Future work will need to assess whether developmental changes in neural variability drive those in behavioral variability. In sum, both, neural and behavioral variability indices might be a more sensitive measure of developmental differences in response inhibition compared to the standard average-based measurements.

## Highlights

• This is the first study to investigate the development of response inhibition focussing on variability.
• We examined intraindividual variability both in stopping latencies and the underlying neural circuitry.
• There were no developmental differences in mean response inhibition, yet clear differences in performance variability.
• This, in turn, was associated with developmental differences in brain signal variability.
• Behavioral and neural variability indices might be a more sensitive measure of developmental differences in inhibition.

## 1. Introduction

Early executive functions (EF's) are critically important for later life outcomes (Moffitt et al., 2011). Due to this, understanding the nature of developmental change in EF's is crucial for devising interventions seeking to improve this important life-skill. One such ability is response inhibition, the ability to withhold prepotent response tendencies in order to respond appropriately to the environment. Developmental studies of response inhibition show inconsistent findings in terms of age-related changes, with some studies showing no difference between children and adults (Schel et al., 2014), and others showing continuing development (Ordaz et al., 2013). Such discrepant findings could be related to a reliance on measures of inhibition that unreliably index age-related changes in response inhibition, in particular the use of mean response times (Band et al., 2003; Logan and Cowan, 1984; Verbruggen and Logan, 2008).

Across cognitive psychology, there has recently been recognition that over-reliance on the mean may lead to misinterpretation of data and erroneous conclusions (Heathcote et al., 1991; Speelman and McGann, 2013). For example, response time distributions may differ between two groups, but these differences may not be reflected as a difference in means (Whelan, 2008). This may lead researchers to conclude that performance does not differ between groups, when it does. Further, key cognitive operations may not be indexed by the mean. Cognitive performance is not stable and this reflects a fluctuating cognitive system (Shalev et al., 2019) (indeed, the mean itself is derived from a distribution of scores, indicating this variability in performance). The degree and manner to which an individual's cognitive system fluctuates may in fact be crucial to understanding their cognitive performance. Consider, for example, the way in which two children differ in how consistently they attend to a class at school. This type of fluctuation in attention may index a feature of the cognitive system that is not necessarily detectable by mean scores, since mean performance may appear the same (Whelan, 2008), yet may crucially impact their learning and subsequent academic performance on that subject. For these reasons, in recent years there has been increased focus on variability measures *per se*, as a meaningful measure of interest.

One area that has seen fruitful use of variability measures is research into attention-deficit hyperactivity disorder (ADHD), a developmental condition characterized by impulsivity, hyperactivity and inattention, and commonly associated with response inhibition deficits (Wodka et al., 2007). Many studies report that ADHD is associated with increased response time variability (Karalunas et al., 2014; Kofler et al., 2013). Measures of variability provide greater clinical and diagnostic specificity than the mean, for example, variability measures are more sensitive than mean response time measures at differentiating individuals with ADHD from controls (Klein et al., 2006; Nigg, 1999), and stimulant medication specifically impacts variability measures (Epstein et al., 2011). Multiple different cognitive and physiological mechanisms have been proposed as underlying response time variability in ADHD (Tamm et al., 2012). Most widely reported is that response time variability may index distractibility and lapses of attention (Adams et al., 2011; Di Martino et al., 2008; Tamm et al., 2012).

At the neural level, the processes underlying behavioral variability are not fully understood (Garrett et al., 2013). One line of research focusses on the relationship between behavioral and neural variability. Brain signals inherently fluctuate (Faisal et al., 2008; Pinneo, 1966). Much like with behavioral variability, in recent years, researchers have begun to investigate neural variability directly, under the assumption that rather than representing noise, variability *per se* may have functional relevance (Garrett et al., 2013). It has been proposed that neural variability increases the ‘dynamic range’ of responsivity, enabling the brain to respond adaptively in a changeable and dynamic environment (Grady and Garrett, 2018). This interpretation is bolstered by the finding that variability across local brain regions is higher during externally-directed tasks, compared with internally-directed ones, since less flexibility is required for tasks directed internally (Grady and Garrett, 2018). In this way, the relationship between neural and behavioral variability has been proposed to be antithetical (Garrett et al., 2018), whereby increased neural variability enables more stable (less variable) behavioral performance (Garrett et al., 2018; Petroni et al., 2018). This is in line with studies in adults that report that BOLD variability increases when engaged in a task (Garrett et al., 2013) and as task difficulty increases (Garrett et al., 2014).

However, the theoretical accounts proposing an antithetical relationship between neural and behavioral variability are largely based on studies in adults. In contrast, studies in children have provided more mixed findings. Children show greater behavioral variability (as indicated by response time measures) (Li et al., 2009; McAuley et al., 2006; Williams et al., 2005, 2007), which decreases with age (Tamnes et al., 2012). Studies report that reduced variability of behavior over childhood is associated with both increased (McIntosh et al., 2008; Mišić et al., 2010) and reduced neural variability (Guassi Moreira et al., 2019). These contradictory findings in childhood may suggest a changing nature of this relationship across development. Notably, this may vary with specific task demands (Armbruster-Genc et al., 2016) and across brain regions (Nomi et al., 2017). Such contradictory patterns of neural variability with age make this an urgent field of further enquiry. In particular, it is important that more developmental studies are conducted investigating this relationship across a range of tasks to gain a comprehensive understanding of the underlying mechanisms. To our knowledge, no study has investigated neural variability in relation to performance variability of response inhibition. Given that response inhibition enables flexible responding to changes in the environment, this makes it a suitable candidate for a behavioral task that may be associated with neural variability. Thus, in the present study we sought to conduct the first investigation of the development of neural and behavioral variability in the context of response inhibition.

Previous studies investigating the neural correlates of variability in response inhibition performance in adults report that ‘Go’ trial variability correlates with commission errors (Bellgrove et al., 2004), and that higher variability is associated with activation of prefrontal regions during ‘No Go’ trials in both adults (Bellgrove et al., 2004) and children (Simmonds et al., 2007). These studies used average BOLD signal, and did not investigate BOLD variability. Further, for performance variability, these studies used measures that assume a Gaussian data distribution. The main outcome variable in response inhibition tasks is response time data, which is characteristically positively skewed and therefore non-Gaussian. Using approaches that assume a normal distribution on non-normal data may lead to loss of information and loss of power (Ratcliff, 1993). Due to this, approaches that do not assume a normal distribution may be more appropriate (Balota and Yap, 2011).

To overcome these limitations, we used an ex-Gaussian distribution in the current study, which is similar to a typical response time distribution (Luce, 1986). Ex-Gaussian distributions convolve the normal with an exponential distribution, generating three parameters, mu (μ) and sigma (σ) reflect the mean and standard deviation of the Gaussian distribution and tau (τ) reflects the mean and standard deviation of the exponential distribution (the tail of the curve). Ex-Gaussian analysis has led to unique insights in other areas (Chevalier et al., 2014; Matzke et al., 2017). For example, a meta-analysis reported that in individuals with ADHD, increased response time variability is primarily related to particularly slow responses on a subset of trials which may relate to attentional lapses, reflected by tau, rather than consistent variability across all trials, reflected by sigma (Kofler et al., 2013).

In this study, we provide the first investigation of the development of response inhibition related BOLD signal variability from childhood to early adulthood and its influence on intra-individual variability in response inhibition performance. Intra-individual variability in response inhibition performance was estimated using Bayesian estimation of ex-Gaussian stop-signal response time distributions (Matzke et al., 2013b). Brain signal variability was estimated during successful stopping. For analyses relating brain signal variability to behavioral response inhibition, we focused on regions frequently implicated in inhibition (Aron et al., 2007; Jahfari et al., 2011), specifically the right inferior frontal gyrus, right caudate nucleus, right putamen, right thalamus, and right subthalamic nucleus, henceforth referred to as the ‘inhibition network’. To examine the relationship between behavioral and brain signal variability, we investigated the correlations between these measures.

We hypothesized that intra-individual variability in response inhibition performance would decrease from childhood to adulthood. Given that there are few studies investigating BOLD variability in development, our investigations regarding changes in BOLD variability from childhood to adulthood and in terms of correlations with performance, were exploratory.

## 2. Methods

### 2.1. Participants

Nineteen healthy right-handed children between 10 and 12 years of age (10 females, *M* = 11.56, *SD* = 0.83) and twenty-six healthy right-handed adults between 18 and 26 years of age (15 females, *M* = 21.55, *SD* = 2.31) participated in the experiment. We did not have any participant exclusion criteria for conditions (such as ADHD or substance use disorders) that may be associated with differences in SSRT performance, as our intention was to sample a cohort that is representative of the general population. The mean behavioral and fMRI results of a subset of the adults have previously been published in a larger report on response inhibition (Schel et al., 2014). A chi-square test revealed no significant differences in gender distributions between age groups (*p* = .736). All participants had normal or corrected-to-normal vision, and no neurological or psychiatric impairments according to self- or parent-report. Informed consent was obtained for all participants and the study was approved by the internal review board at Leiden University Medical Center. In accordance with the guidelines of the Leiden University Medical Center, all anatomical scans were reviewed by a radiologist. No anomalous findings were reported.

To obtain an estimate of cognitive functioning, children and adults completed the subtests similarities and block design of the Wechsler Intelligence Scale for Children (WISC) (Wechsler, 1981b) and the Wechsler Adult Intelligence Scale (WAIS) (Wechsler, 1981a) respectively. Estimated IQ scores were slightly above average (children: *M* = 111.32, *SD* = 9.94, adults: *M* = 111.81, *SD* = 6.59) and age groups did not differ in estimated IQ scores, *F* (1, 44) = 0.04, *p* = .843.

### 2.2. Task

The stop-signal task (Logan and Cowan, 1984) was presented in a visual form (Schel et al., 2014). Each trial started with the presentation of a green left- or rightwards pointing arrow. Participants were instructed to make a speeded response to the direction of the arrow. For the leftwards pointing arrow participants had to press a button with their left index finger and for the rightwards pointing arrow participants had to press a button with their right index finger. The arrow disappeared when participants responded or after 1500 milliseconds had passed. Following the presentation of the arrow a fixation cross was presented with a duration jittered between 2000 and 4000 milliseconds. When participants responded to the arrow, the duration of the fixation cross was extended by 1500 milliseconds minus the response time, in order to keep the duration of the task stable between participants.

On a limited number of trials (25%) a stop-signal was presented. In this case, the arrow suddenly changed color to red. This color change indicated that participants had to inhibit responding to the direction of the arrow. Stop-signal delay (SSD) was adjusted using a staircase-tracking procedure to guarantee a 50% inhibition rate (Lappin and Eriksen, 1966). At the beginning of the task SSD was set at 250 milliseconds. When participants successfully inhibited, SSD was increased by 50 milliseconds to make the task more difficult. When participants were not able to inhibit responding, SSD was decreased by 50 milliseconds to make the task easier.

The experiment consisted of two blocks of 128 trials, each block consisting of 96 go-trials and 32 stop-trials. Trials were presented in a pseudo-randomized order so that each stop-trial was preceded by 1, 2, 4, or 5 go-trials.

For this task, we used an exclusion criterion of 30% successful stops. This was checked for all participants in order to exclude participants with less than this. No participants were excluded on this basis.

### 2.3. Data acquisition

Scanning was performed with a standard whole-head coil on a 3.0 Tesla Philips scanner at the Leiden University Medical Center. The stop-signal task consisted of 2 event-related runs, both lasting approximately 5 min. Functional data were acquired using T2*-weighted echo-planar imaging (EPI). The first 2 vol. of each run were discarded in order to allow for equilibration of T1 saturation effects (TR = 2.2 s, TE = 30 ms, sequential acquisition, 38 slices of 2.75 mm, field of view 220 mm, 80 × 80 matrix, in-plane resolution 2.75 mm). After the functional runs, a high-resolution 3D T1-FFE scan for anatomical reference was obtained (TR = 9.760 ms, TE = 4.59 ms, flip angle = 8°, 140 slices, 0.875 × 0875 × 1.2 mm^3^ voxels, field of view = 224 × 168 × 177 mm^3^). Head motion was restricted by using foam inserts between the head and the head coil. Visual stimuli were projected onto a screen in the magnet bore that could be viewed through a mirror attached to the head coil.

### 2.4. Behavioral data analysis

We first used a canonical calculation of SSRT and secondly calculated measures of SSRT variability.

*Calculation of SSRT.* The SSRT was calculated according to the horse-race model of stopping (Logan and Cowan, 1984) following the procedures described in Band et al. (2003). In short, first all response times (RTs) for the correct go-trials were rank-ordered. Next, the percentage of failed inhibitions was determined. Then, the go-RT corresponding to that percentage was determined. Finally, SSRT was computed as the difference between the go-RT corresponding to the percentage of failed inhibitions and the mean SSD.

*Calculation of SSRT variability.* In order to estimate the intra-individual variability in SSRTs, the entire distribution of SSRTs was estimated using a Bayesian Parametric Approach (BPA) (Matzke et al., 2013a, 2013b). The BPA assumes that SSRTs form an ex-Gaussian distribution and uses Markov chain Monte Carlo (MCMC) sampling of the observed participant SSRT data in order to estimate the three parameters that describe the SSRT distribution (Matzke et al., 2013b). These parameters describe different parts of the SSRT curve: the μ and σ reflect the mean and standard deviation of the curve and the τ reflects the skewness (tail) of the curve. The validity of the BPA approach in estimating SSRT distributions has been demonstrated through parameter recovery studies on both real and simulated data, which demonstrate that the BPA approach accurately recovers underlying parameters from the SSRT distribution (Matzke et al., 2013a). BEESTS software version 2.0 (Matzke et al., 2013b) was used to implement the BPA on the hierarchical child stop-signal data and the hierarchical adult stop-signal data. The hierarchical approach simultaneously estimates the group level parameters as well as the participant level ‘go’ and ‘stop’ parameters. This is especially valuable when there is a small number of observations and moderate between subject variability in parameter values (Matzke et al., 2013b). The following settings were used for the MCMC sampling: number of chains = 3, number of samples = 30,000, number of burn-in = 10,000, amount of thinning = 10, number of predictions = 1000, and trigger failure = TRUE. The trigger failure setting was used to allow for identifying deficiencies in detecting the stop-signal, which can distort estimation of the SSRT distribution when ignored (Matzke et al., 2017).

### 2.5. FMRI data analysis

*Data preprocessing.* Data were preprocessed using SPM12 (Wellcome Department of Cognitive Neurology, London) and the CONN toolbox (Whitfield-Gabrieli and Nieto-Castanon, 2012). The standard preprocessing pipeline in CONN, including slice-time and motion correction, normalization to the MNI template, resampling to a 3-mm cubic voxel, and smoothing with an 8-mm full-width-at-half-maximum isotropic Gaussian kernel was applied. To further denoise the data for the BOLD variability calculation, denoising in CONN was conducted to remove physiological artifacts and residual movement effects. Specifically, average time series of white matter, cerebral spinal fluid (CSF), realignment parameters, and volumes affected by motion as detected by Artifact Detection Tools (ART) were filtered out. These extra denoising steps have been shown to improve the predictive power of BOLD variability (Garrett et al., 2010; Garrett et al., 2014).

*Calculation of BOLD variability.* BOLD signal variability was calculated using SPM 8 by calculating the difference of residuals (DoR) of two different regression models. All steps undertaken are in line with the procedures described in Armbruster-Genc et al. (2016). In short, first a standard regression model using a general linear model (GLM) with a canonical hemodynamic response function (HRF), one regressor per condition, one regressor for the error trials and the time and dispersion derivatives to account for variability in peak and width of the HRF was estimated. Next, a condition specific trial-by-trial regression model was estimated. This model was similar to the standard model, except that for the condition of interest a separate regressor for each trial of that condition was added to the model, instead of the one condition regressor. By subtracting the residuals of the standard regression model from the residuals of the condition specific trial-by-trial model, an estimate of the trial-by-trial variability in one specific condition is obtained. The DoR was calculated for the successful stop condition. Importantly, the residuals maps used for the subtraction were not corrected for degrees of freedom of the regression model, since the degrees of freedom of the condition specific trial-by-trial regression model are confounded with the number of successful versus unsuccessful stop-trials which could differ between participants. Correcting for degrees of freedom would therefore bias the DoR of subjects with different ratios of successful versus unsuccessful stop-trials (Armbruster-Genc et al., 2016). Finally, individual DoR images were entered into a second-level analysis to examine developmental differences in condition specific BOLD variability. All reported effects consisted of at least 10 contiguous voxels that exceeded a family-wise error (FWE) corrected threshold of *p* < .05.

*Region of interest analysis.* Region of interest (ROI) analysis were performed to further characterize the BOLD variability of the inhibition network using the MarsBaR toolbox in SPM (Brett et al., 2002) (http://marsbar.sourceforge.net). For right caudate, right putamen, and right thalamus anatomical AAL ROIs were selected from the MarsBaR-AAL ROIs (centers of mass: right caudate: 15, 11, 8; right putamen: 28, 4, 1; right thalamus: 13, −19, 7). An anatomical ROI of right IFG pars triangularis was selected from the probabilistic Harvard-Oxford atlas and thresholded at 20%, center of mass: 51, 28, 8. For right subthalamic nucleus (STN), an anatomical template derived from a study using ultra-high 7 Tesla scanning was used (Forstmann et al., 2010), center of mass: 9, −13, −7. BOLD variability was extracted both from the separate ROI's (for the individual ROI analyses) and as an average from the entire ROI network (for the network analysis). To test the specificity of the relationship between inhibition network BOLD variability and behavioral variability, we also extracted BOLD variability across a control region. We selected the right calcarine cortex as a control region, as this region has not been implicated in response inhibition performance and thus we would not hypothesize there to be any relationship between neural variability in the calcarine cortex and response inhibition variability.

### 2.6. Experimental design and statistical analysis

Age group differences in average response inhibition performance, response inhibition performance variability and brain signal variability were tested using ANOVA's. A threshold of *p* < .05 was used. To examine the relation between behavioral and brain signal variability Pearson correlation analysis was performed. Z observation analysis was used to determine differences in Pearson's correlations between groups. Subject motion in the scanner is an important consideration when assessing between-group differences, including for variability measures (Millar et al., 2020). Therefore, to rule out any effect of between-group differences in head motion, we conducted an independent *t*-test on the amount of motion-correction applied during processing. In order to do this, mean framewise displacement data was extracted. Further, to test whether BOLD variability was associated with in scanner motion, we tested whether mean framewise displacement was correlated with BOLD variability in the inhibition network.

### 2.7. Power

We conducted a power analysis based on effect size estimates from previous studies to determine whether the present study has enough power to detect the effects of interest. We based this power analysis specifically on prior studies that have looked at age-related changes in BOLD variability (Garrett et al., 2010; Grady and Garrett, 2018) including in the context of cognitive control (Grady and Garrett, 2018). Grady and Garrett (2018) report an age-group comparison of BOLD variability. The effect size in this study was 1.2 which is considered very large according to the criteria of Cohen (Cohen, 1988). With the power to detect this effect at 0.80 and an alpha of 0.05, the projected sample size needed to detect such an effect is 24, with 12 in each group. An additional study reports a very large effect size (4.26) associated with the relationship between age and BOLD variability (Garrett et al., 2010). With the power to detect a similar effect set at 0.80 and an alpha of 0.05, 7 subjects would be required to detect such an effect. Based on this, we consider this study to be sufficiently powered to detect the age-related differences in BOLD variability.

## 3. Results

### 3.1. Behavior

**Fig. 1:**
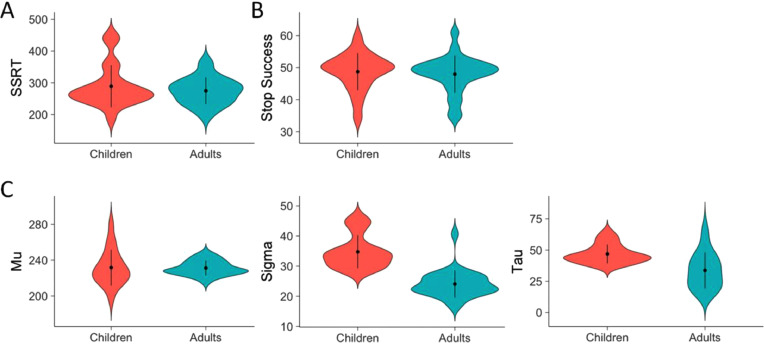
**A.** Mean stop-signal reaction time (SSRT) per age group. **B.** Percentage stop success per age group. **C.** Measures of variability: mu (mean), sigma (standard deviation), and tau (skewness or tail) per age group. Means (dot) and standard error (line) are presented.

**Fig. 2:**
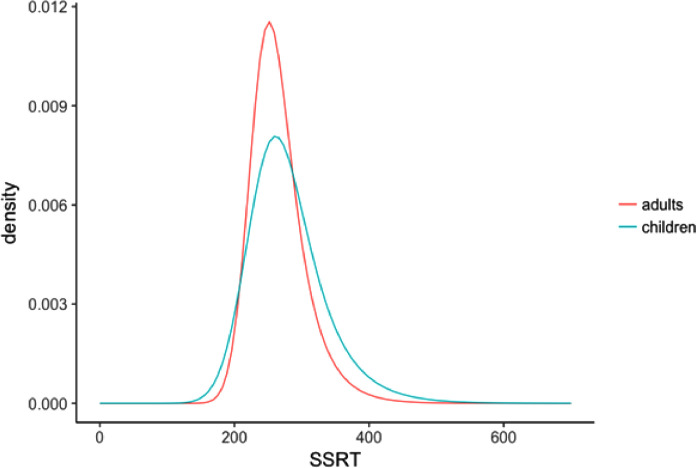
Estimated SSRT distributions for children and adults.

Mean SSRT did not differ between children and adults, *F* (1, 44) = 0.799, *p* = .376 (Fig. 1A). Children and adults did not differ in the percentage of successful stop-trials, *F* (1, 44) = 0.185, *p* = .669, and both successfully inhibited approximately 50% of the stop-trials (Fig. 1B). Next, in order to examine developmental differences in intra-individual variability in stopping performance, the estimated SSRT distributions and the three parameters describing the SSRT distribution were examined. The estimated SSRT distributions differed between children and adults, with children showing a wider distribution driven by more extremely long SSRTs (Fig. 2). The μ did not differ between children and adults, *F* (1, 44) = 0.013, *p* = .911, (Fig. 1C) indicating that the mean of distribution is similar for both groups. However, σ was larger for children compared to adults, *F* (1, 44) = 51.388, *p* < .001, η^2^ = 0.54, (Fig. 1C) indicating that the SSRT distribution of children is wider. Also, τ was larger for children compared to adults, *F* (1, 44) = 13.026, *p* = 0.001, η^2^ = 0.23 (Fig. 1C) indicating that the SSRT distribution of the children had a longer tail and thus children showed more extreme long SSRTs.

### 3.2. BOLD variability

**Fig. 3:**
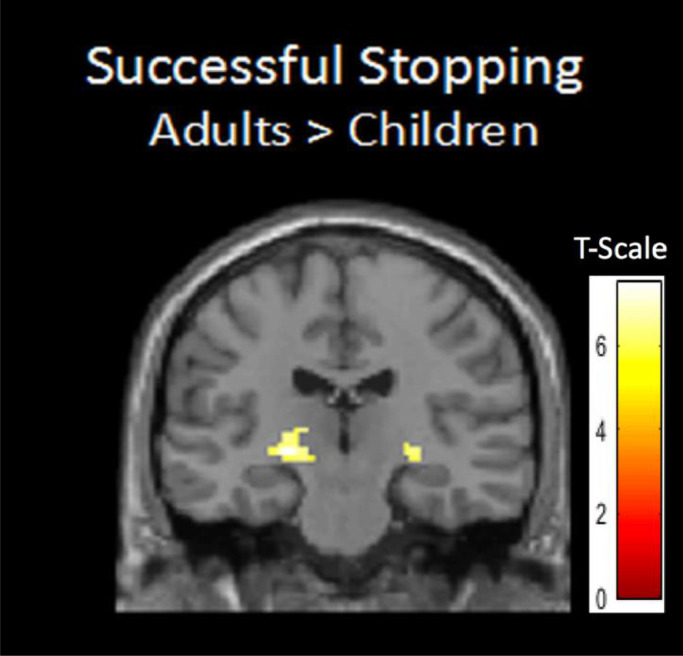
Adults showed more BOLD variability compared to children in bilateral thalamic area during successful stopping.

To examine age differences in BOLD variability during successful stopping a whole-brain two sample *t*-test was computed. This analysis revealed that BOLD variability in bilateral thalamic area was larger for adults compared to children (left peak: −20, −22, −4, t(43) = 7.50, *p* < .001 and right peak: 28, −22, −4, t(43) = 6.24, *p* = .007, see Fig. 3). No regions showed more BOLD variability for children compared to adults.

**Fig. 4:**
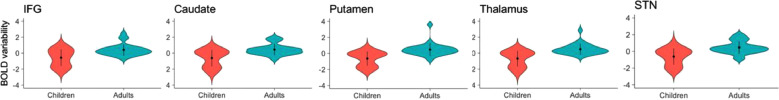
Age differences in standardized BOLD variability levels per region. Adults showed more BOLD variability compared to children in all regions of interest during successful stopping. Means (dot) and standard error (line) are presented.

To further examine BOLD variability during successful stopping, ROI analyses were performed for the right IFG pars triangularis, right caudate, right putamen, right thalamus, and right STN. Adults showed more BOLD variability compared to children in all ROIs during successful stopping (right IFG: *F* (1, 44) = 13.60, *p* = 0.001, η^2^ = 0.24, right caudate: *F* (1, 44) = 16.89, *p* <0.001, η^2^ = 0.28, right putamen: *F* (1, 44) = 19.57, *p* <0.001, η^2^ = 0.31, right thalamus: *F* (1, 44) = 23.03, *p* <0.001, η^2^ = 0.35, right STN: *F* (1, 44) = 17.60, *p* <0.001, η^2^ = 0.29) (see Supplementary materials Table 1 and Fig. 4).

We also tested whether movement parameters during the scan were related to BOLD variability in the inhibition network. Mean framewise displacement did not differ significantly between children (*M* = 0.22, *SD* = 0.09) and adults (*M* = 0.19, *SD* = 0.04); t(43)=1.59, *p* = .120. There was no significant correlation between framewise displacement and BOLD variability in the inhibition network for either the whole group, *r* = −0.113, *p* = .458, or for the children and adults separately (child: *r* = 0.004, *p* = .987; adult: *r* = 0.087, *p* = .674). Scatterplots of these correlations are included in the Supplementary materials.

### 3.3. Correlations between neural and behavioral variability

**Fig. 5:**
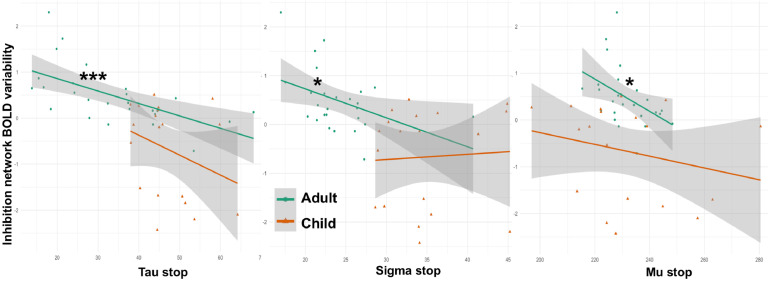
Correlations between BOLD variability across the inhibition network during successful stopping and behavioral performance variability, as measured by tau, sigma and mu.

To examine the relation between BOLD variability and behavioral performance variability, correlation analyses were performed for the age groups separately. As can be seen in Fig. 5, more BOLD variability across the inhibition network during successful stopping was associated with less behavioral variability, specifically with less extremely long SSRTs (tau) and a less wide SSRT distribution (sigma) and smaller SSRTs on average. The relationship between BOLD and behavioral ex-Gaussian measures was significant for the adult group (tau: *r* = −0.614; *p* < .001; sigma: *r* = −0.421; *p* < .05; mu: *r* = −0.439; *p* < .05). In children, the direction of effect was similar, but the correlations did not reach significance (tau: *r* = −0.317; *p* = .186; sigma: *r* = −0.057; *p* = .818; mu: *r* = −0.244, *p* = .316). There were no significant differences between groups on any of these correlations, when tested using a *Z* observation analysis.

To test the specificity of the relationship between BOLD variability during successful inhibition and SSRT variability we also calculated the ex-Gaussian measures (tau, sigma and mu) for the ‘Go’ trials and tested the relationship between these parameters and BOLD variability. We found no significant relationship between any of these parameters for either group. Finally, we included the ‘Go’ trial ex-Gaussian parameters as control variables in partial correlations between stopping variability and BOLD variability. We found that the significance of these relationships remained unchanged for both the adults (tau: *r* = −0.696; *p* < .001; sigma: *r* = −0.46; *p* < .05; mu: *r* = −0.430, *p* < .05) and children (tau: *r* = −0.270; *p* = .31; sigma: *r* = −0.139; *p* = .6; mu: *r* = −0.149, *p* > .05).

Finally, to investigate whether the reported relationship between inhibition network BOLD variability and behavioral variability is specific to the right inhibition network, correlation analyses were performed between behavioral variability and BOLD variability measures across the control region, for the groups separately. We did not find any significant correlations in either group (adult group, tau: *r* = −0.28; *p* > .05; sigma: *r* = −0.02; *p* > .05; mu: *r* = −0.29, *p* > .05; child group, tau: *r* = −0.35; *p* > .05; sigma: *r* = 0.06; *p* > .05; mu: *r* = −0.31, *p* > .05).

## 4. Discussion

In contrast to previous developmental neuroimaging studies of response inhibition, we were specifically interested in behavioral and brain signal variability during response inhibition. As predicted, children were more variable in their response inhibition performance compared to adults, while brain signal variability in the inhibition network was higher for adults. Brain signal variability was negatively related to behavioral variability in the adult group. This effect was specific to the stop-signal and was present across the inhibition network, but not in the control region we investigated.

The first question addressed whether children would show more intra-individual variability in response inhibition performance compared to adults. Previous research has shown that children show increased response time variability (Li et al., 2009; McAuley et al., 2006; Williams et al., 2005, 2007), and that variability decreases with age (Tamnes et al., 2012). However, developmental intra-individual differences in SSRTs have not been studied. Despite the absence of average performance differences, we found that children were more variable in their response inhibition performance, with children showing a wider distribution and more extremely long SSRTs. Extremely long SSRTs can be caused by lapses in attention (Di Martino et al., 2008).

Our findings are in line with the observation that in childhood intra-individual variability in response inhibition decreases with practice (Chevalier et al., 2014). With the current cross-sectional dataset, we cannot disentangle whether this decreased intra-individual performance variability with age is caused by a maturational effect of the underlying neural mechanism, a practice effect of increased application of response inhibition strategies with age, or a combination of those factors. The observation of more intra-individual variability together with similar average performance in children compared to adults is interesting since this implies that in addition to showing more extremely long SSRTs, children must also show more fast stopping responses in order to end up at the same average performance as adults. Alternatively, this effect could be explained by slower “Go” responses. It will be important for future research to examine this.

The next question addressed whether response inhibition related brain signal variability would increase from childhood to adulthood. The results showed that BOLD variability was higher in adults compared to children during successful stopping, with the bilateral thalamic region showing significant group differences on a whole-brain level. Importantly, the observed age-related increase in brain signal variability was significant across the inhibition network, assessed via the ROI analysis.

The observation of higher task-related brain signal variability in adults compared to children is in line with previous research examining task-related EEG and MEG brain signal variability (McIntosh et al., 2008; Lippe, 2009; Misic, 2010). However, our results are seemingly at odds with recent developmental studies investigating BOLD variability (Guassi Moreira et al., 2019; Montez et al., 2017; Nomi et al., 2017). Nomi et al. (2017) report that resting state BOLD variability increased with age across the salience network, but decreased across all other brain regions, including the thalamus, for which we found a strong developmental effect in the current study. An important distinction between the current findings and those reported by Nomi et al. (2017), may be the nature of neural variability when comparing resting state and task-based designs such as ours, particularly considering that neural variability increases from rest to task (Garrett et al., 2013a). Yet, other developmental imaging studies using task based designs, also report results that appear to contrast with ours, for example that neural variability decreases with age when using tasks assessing emotion regulation (Guassi Moreira et al., 2019) and working memory (Montez et al., 2019).

That our findings appear to contrast with other developmental imaging studies point towards important areas within the field of variability that still require clarification – in particular, to what degree is neural variability functional, and under what circumstances (Dinstein et al., 2015; Garrett et al., 2013)? The idea that neural variability has a functional role, rather than being ‘noise’, may best be understood in the context of the brain needing to respond adaptively to circumstances that are changing and unpredictable (Garrett et al., 2013). In this case, a deterministic or rigid neural response pattern may not be advantageous. Yet, excessive levels of instability may also be detrimental, and have been associated with behavioral impairments in neurodevelopmental disorders such as ADHD and autism (Karalunas et al., 2014). It appears that there may be an optimal level of variability, with both too little and too much potentially being suboptimal (Dinstein et al., 2015). It may also be the case that the ‘optimal’ level of neural variability differs, depending on the context and precise requirements of the activity. For example, Armbruster-Genc et al. (2016) report that neural variability in the inferior frontal junction is associated with better or worse performance depending on the task. Specifically, higher neural variability was associated with higher cognitive flexibility performance, but lower performance on a cognitive stability task. This highlights the importance of task specifics, when seeking to understand the nature of neural variability in relation to performance. Task differences may help to explain the discrepancy between our findings and other task-based studies (Guassi Moreira et al., 2019; Montez et al., 2017). As this study is, to our knowledge, the first to investigate neural variability and its relation to response inhibition, further studies will be required to disentangle these relationships.

Importantly, brain signal variability during successful inhibition was also associated with stopping performance in the adult group, in particular, variability of the ‘stop’ but not ‘go’ trials. This was seen across the inhibition network but not the control region analyzed. Together, these points highlight the considerable specificity of our observed effects. This is the first study to report that decreased stopping response time variability is associated with heightened BOLD signal variability across regions of the right lateralized inhibition network. Previous studies investigating the neural basis of response inhibition variability have reported that higher behavioral variability is associated with higher mean prefrontal activation (Bellgrove et al., 2004; Simmonds et al., 2007), whilst individuals with lower behavioral variability activated premotor regions (Simmonds et al., 2007).

There are several important distinctions between these previous studies and our own.

Firstly, previous studies used a variability measure of the response selection (‘go’) trials; in contrast, this is the first study to investigate variability of stopping trials, which (although not a direct measure), may be considered a purer measure of inhibition ability. Secondly, the variability index used in previous studies (the intra-individual coefficient of variability) is based on the Gaussian distribution. As response time distributions are characteristically non-Gaussian, using methods with Gaussian assumptions on such data may lead to loss of power and inappropriate conclusions (Balota and Yap, 2011; Ratcliff, 1993). In contrast, in the present study we used ex-Gaussian measures, which may be more appropriate as they enable full characterization of the response time distribution, and may be more sensitive (McAuley et al., 2006). Finally, previous studies used average BOLD signal. As it has been demonstrated that mean BOLD activity and BOLD variability are independent (Garrett et al., 2011) this points to an interesting distinction between the functionality of the two measures, and highlights the importance of further investigating this relationship in more studies.

A potential mechanism through which brain signal variability could influence behavioral variability is through increased connectivity with the rest of the brain (Burzynska et al., 2015; Garrett et al., 2018). When activity in a brain region is more variable this could allow for more adaptability and synchronization with other relevant brain areas and thereby result in less variable and thus better behavioral performance (Tamnes et al., 2012). Indeed, local brain signal variability is related to stronger network integration (Garrett et al., 2018). This is in line with the observed relation between brain signal variability and white matter integrity (Burzynska et al., 2015), suggesting that increased structural connectivity, facilitated by synaptic connections and structural properties such as myelination, may underpin network connectivity and enable greater brain signal variability. Importantly, the thalamus, the only region for which we found a developmental effect on the whole brain level, appears to play a key role here; stronger local brain signal variability in the thalamus, is related to greater network integration in adults (Garrett et al., 2018).

Our report of extremely long SSRTs in the child group, suggestive of attentional lapses (Di Martino et al., 2008), may relate to neural variability related network integration. Attention is maintained by competition between task-based and task-free (default mode) brain networks (Hopfinger et al., 2000), the interaction of which is underpinned by structural connectivity (Shen et al., 2015). Performance that is more stable (Kelly et al., 2008) and attention that is ‘in the zone’ is associated with greater anti-correlation between these networks (Kucyi et al., 2017). Anti-correlation between the task-based and default mode networks may be less efficient if networks are less well integrated. This may suggest that increased lapses in attention in the child group may relate to the reported lower levels of neural variability, as this may be indicative of lower levels of network integration (Garrett, 2018). As we do not directly report on functional connectivity in the present study, it will be informative for future studies to directly investigate whether functional connectivity does indeed underpin the reported effects presented.

Some limitations of the present study deserve mentioning. In particular, the sample size included in the present study is relatively small. Whilst such a sample size may be acceptable for the group comparisons which form the main component of our findings, it does warrant caution when considering the reported correlations. Specifically, whilst we report a significant relationship between brain variability and behavioral performance in the adult group, in the child group this relationship did not reach significance. We are not able to conclusively state whether this suggests that the degree of the relationship is different across the groups (indeed, we did not find a significant difference in the degree of relationship, when directly compared using a *Z* observation), since it is possible that the relationship in the child group may not reach significance for other reasons, such as lack of power. Although the power analysis that we conducted based on previous studies suggested that we are likely to have had sufficient power with the sample size included in this sample, it is important to note that, due to the small number of previous studies investigating such effects, our power analysis was based on a small number of studies, themselves with small samples, which may be associated with publication bias and may lead to power overestimation. Thus, it will be necessary for future studies to investigate these relationships in larger samples. Finally, our intention in the present study was to include brain regions that are typically activated during response inhibition. We focused on inferior frontal and basal ganglia regions as these are regions that have both commonly been associated with response inhibition and have previously been investigated in the context of neural variability (Garrett et al., 2018). However, additional brain regions that we did not investigate here may also play a role in response inhibition. Future studies will be required to establish whether the findings reported extend to other regions, such as the pre-supplementary motor area, which were not included in the present study.

An important avenue for future research will be to assess whether it is possible to stimulate children to perform less variably. Cognitive training seeking to improve the consistency of performance may be associated with distinct performance benefits, irrespective of whether there is also an associated change in the mean. At present, this suggestion is speculative as cognitive training studies typically focus on improving mean performance and little is known about whether it is possible to specifically improve performance variability and if so, whether this is associated with specific transfer effects (Smid et al., 2020).

To conclude, the present study is the first to examine behavioral and neural variability during response inhibition from a developmental perspective. Whilst children performed at adult level when looking at average measures, children's response inhibition performance was more variable. In contrast, adult brain signal variability was higher than in children. Furthermore, increased brain signal variability in regions of the inhibition network in adults was associated with reduced performance variability. This may suggest that variability in stopping performance is increasingly associated with neural variability in the inhibition network in adulthood. Together these results underscore the importance of examining behavioral and neural intra-individual variability measures. Neural and behavioral variability indices might be a more sensitive measure of developmental differences compared to the standard average-based measurements and changes in executive functions might be best understood in terms of performance consistency.

## Data statement

It is not possible for the data to be made openly available due to fact that at the point of collection permission was not given by participants for their data to be shared.

## CRediT authorship contribution statement

**Abigail Thompson:** Writing - review & editing, Formal analysis, Visualization. **Margot A. Schel:** Conceptualization, Methodology, Formal analysis, Investigation, Writing - original draft, Visualization. **Nikolaus Steinbeis:** Conceptualization, Methodology, Supervision, Project administration, Funding acquisition, Writing - review & editing.

## Declaration of Competing Interest

None.

## Appendix Supplementary materials

Image, application 1
