# Supplementary material for: Changes in BOLD variability are linked to the development of variable response inhibition
Source: Neuroimage. 2021 Mar;228:117691. doi: 10.1016/j.neuroimage.2020.117691 (PMC7903157; doi:10.1016/j.neuroimage.2020.117691)
Supplement: Supplementary file 1 [file mmc1.docx]

Table 1 Region of interest BOLD variability measures during successful stopping. Means (sd).

|  |  |  |  |
| --- | --- | --- | --- |
|  | Child (N = 19) | Adult (N = 26) | p-value |
|  |  |  |  |
| Inhibition network | -.67 (1.03) | .48 (.64) |  |
| IFG | -.57 (1.05) | .41 (.74) | < .001 |
| Caudate | -.61 (1.04) | .45 (.69) | < .001 |
| Putamen | -.65 (.89) | .47 (.80) | < .001 |
| Thalamus | -.68 (.98) | .50 (.68) | < .001 |
| STN | -.62 (.99) | .46 (.74) | < .001 |

* p < 0.05, ** p < 0.01, *** p < 0.001. Abbreviations: IFG, inferior frontal gyrus; STN, subthalamic nucleus. All regions-of-interest are right hemisphere only.


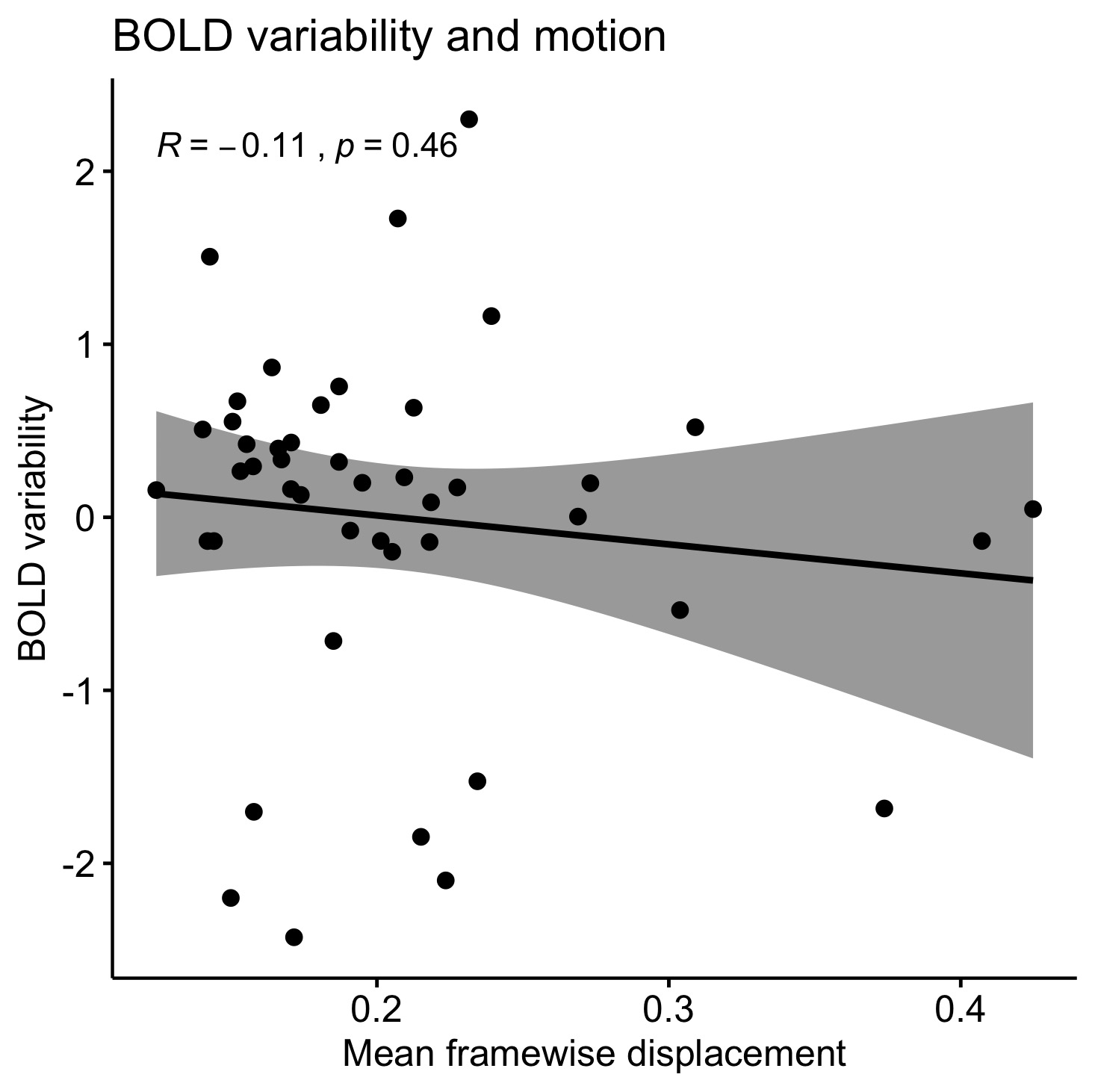


Supplementary Figure 1 Correlation between mean framewise displacement and BOLD signal variability across the inhibition network, across the whole group.


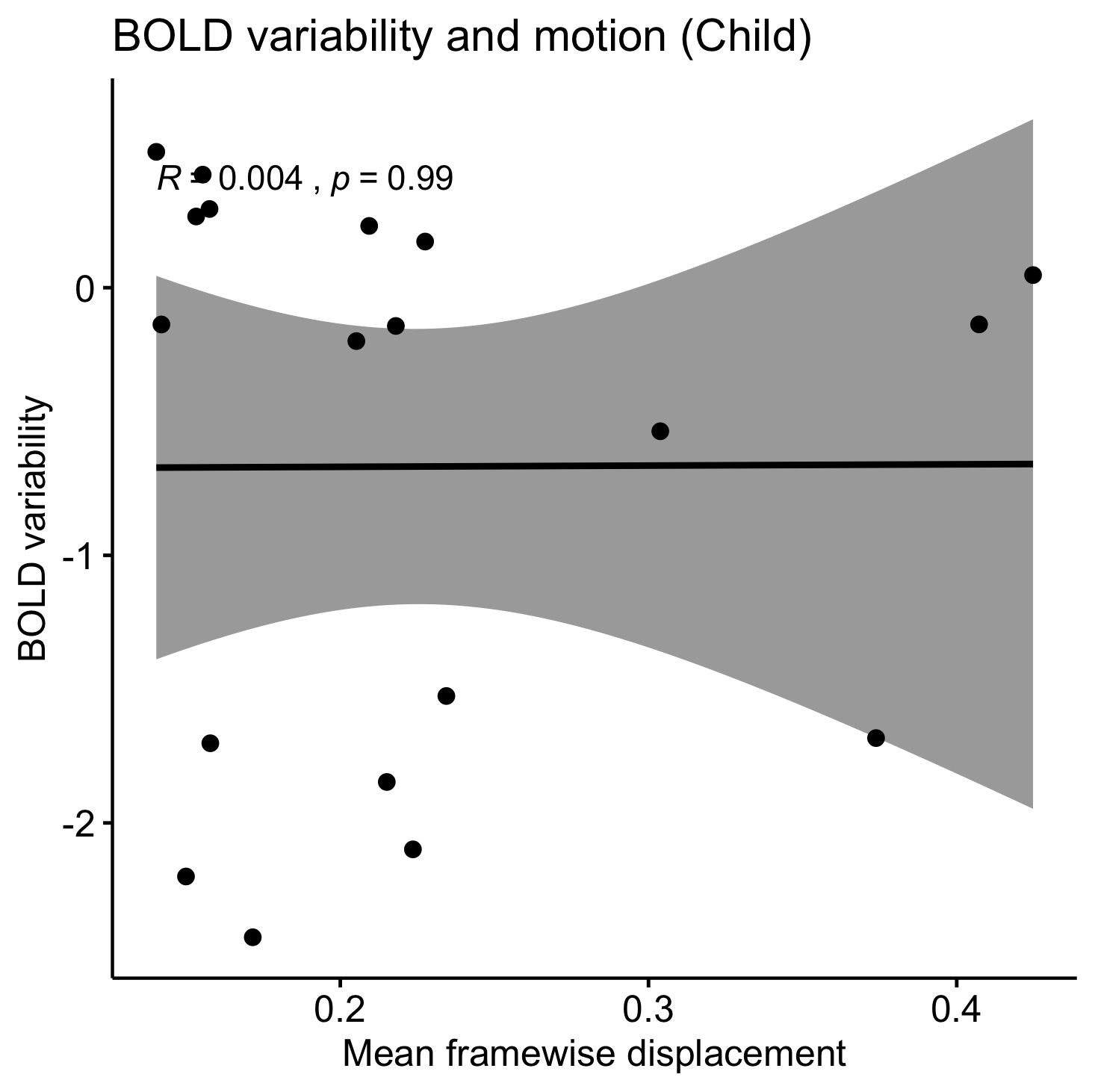


Supplementary Figure 2 Correlation between mean framewise displacement and BOLD signal variability across the inhibition network, child group only.


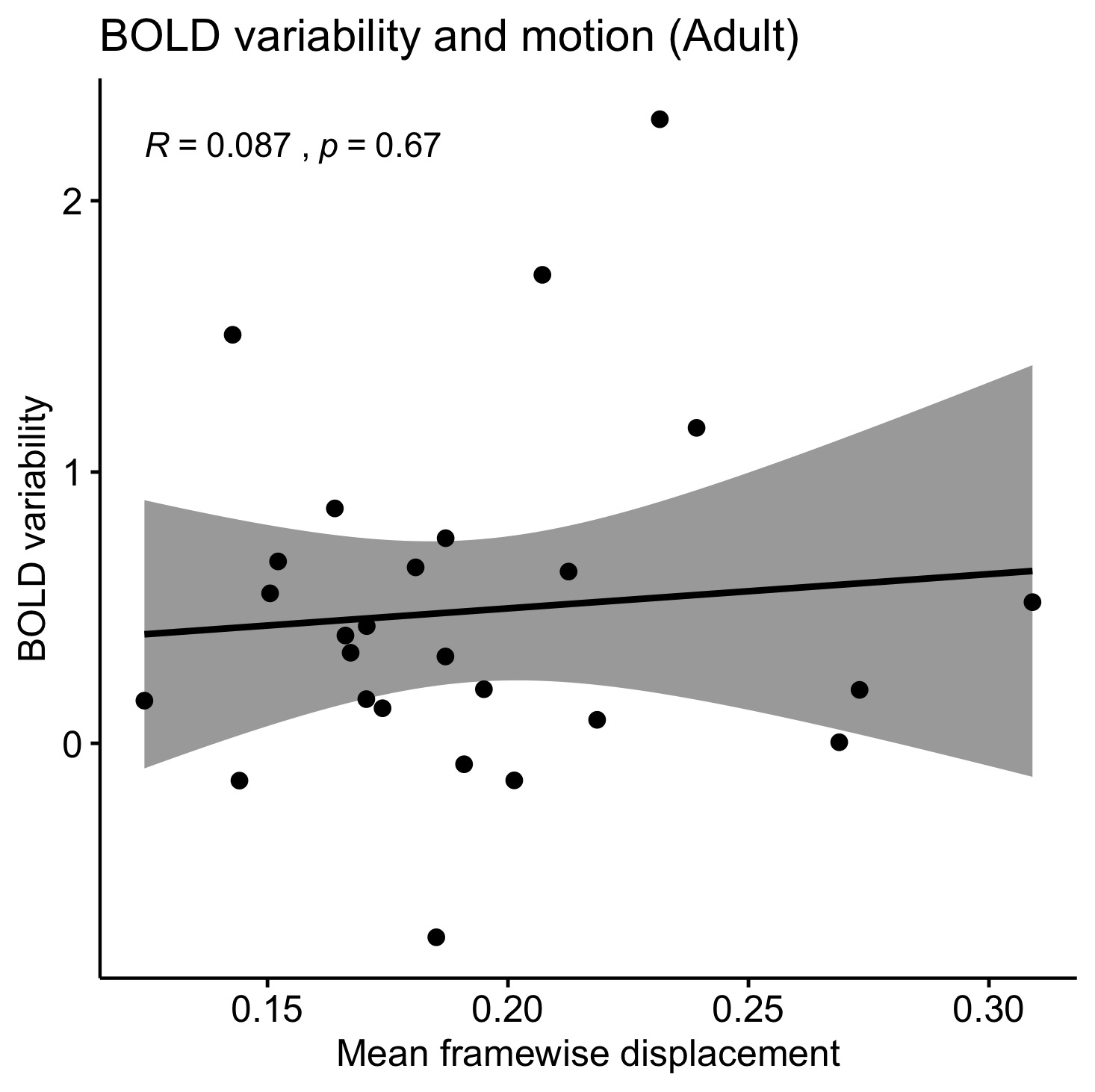


Supplementary Figure 3 Correlation between mean framewise displacement and BOLD signal variability across the inhibition network, adult group only.
